# Registry-Dependent Peeling of Layered Material Interfaces: The Case of Graphene Nanoribbons on Hexagonal Boron Nitride

**DOI:** 10.1021/acsami.1c09529

**Published:** 2021-09-05

**Authors:** Wengen Ouyang, Oded Hod, Michael Urbakh

**Affiliations:** † Department of Engineering Mechanics, School of Civil Engineering, Wuhan University, Wuhan, Hubei 430072, China; ‡ Department of Physical Chemistry, School of Chemistry, The Raymond and Beverly Sackler Faculty of Exact Sciences and The Sackler Center for Computational Molecular and Materials Science, Tel Aviv University, Tel Aviv 6997801, Israel

**Keywords:** peeling, misfit angle, interfacial friction, graphene nanoribbon, h-BN, registry-dependent interlayer potential

## Abstract

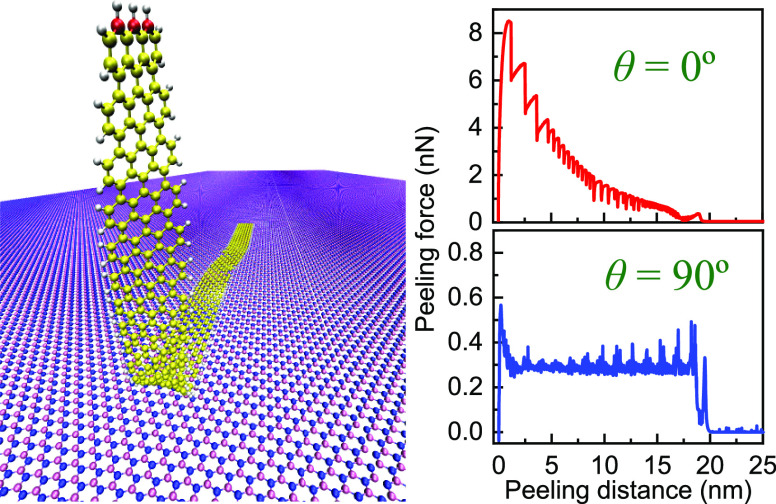

Peeling of layered materials from supporting substrates, which is central for exfoliation and transfer processes, is found to be dominated by lattice commensurability effects in both low and high velocity limits. For a graphene nanoribbon atop a hexagonal boron nitride surface, the microscopic peeling behavior ranges from stick-slip, through smooth-sliding, to pure peeling regimes, depending on the relative orientation of the contacting surfaces and the peeling angle. The underlying mechanisms stem from the intimate relation between interfacial registry, interlayer interactions, and friction. This, in turn, allows for devising simple models for extracting the interfacial adhesion energy from the peeling force traces.

## Introduction

Peeling is an important and ubiquitous process appearing across many length scales ranging from macroscale adhesive tapes^1−3^ and textured materials used in paint, coating, and transfer printing technology;^4,5^ through microscale biological system, such as the toe pads of ants^6^ and geckos;^7−9^ down to nanoscale van der Waals (vdW) materials, such as graphene, hexagonal boron nitride (h-BN), and transition-metal dichalcogenides.^10−13^ It is well known that the latter has unique electronic,^10,14,15^ mechanical,^16−18^ and frictional properties^19−28^ that are best expressed in high-quality single- or few-layered samples. To isolate such samples from their as-grown layered assemblies on various substrates, mechanical exfoliation and transfer remain the simplest and most powerful techniques,^10−12^ in which peeling plays a central role. Thus, it is essential to understand the microscopic nature of the peeling mechanism and find ways to control it.

According to macroscopic intuition, one knows that increasing the peeling angle reduces the resistance of an elastic tape when being removed from a rough surface. This phenomenon is well explained by a simple peeling model based on continuum theory, previously proposed by Kendall.^29^ Since then, a variety of peeling models have been proposed and studied extensively in the context of biological systems.^8,9,30,31^ Understanding peeling processes in nanoscale material junctions, however, requires a detailed atomistic description. In this respect, recent atomic force microscopy (AFM) experiments and molecular dynamics (MD) simulations^32−40^ of the peeling of graphene from various substrates exhibited rich dynamics, which was found to depend on the size, edge structure, and stacking orientation of the peeled graphene flake relative to the substrate, as well as on the external load. In particular, peeling under superlubric conditions (a state of ultralow friction and wear) has been studied via careful experiments and MD simulations of the detachment dynamics of graphene nanoribbons (GNRs) from gold surfaces.^41−45^ Nevertheless, gaining full understanding and control over the peeling process requires the consideration of nonsuperlubric conditions, often encountered in nanomanipulation scenarios. An excellent candidate to study this regime is the peeling of GNRs from h-BN substrates. Due to their small lattice mismatch (∼1.8%), these systems may exhibit stick-slip or smooth-sliding behavior, depending on the relative orientation between the slider and the substrate.^46^ This, in turn, allows for studying various peeling mechanisms using a single platform.

## Methods

**Figure 1:**
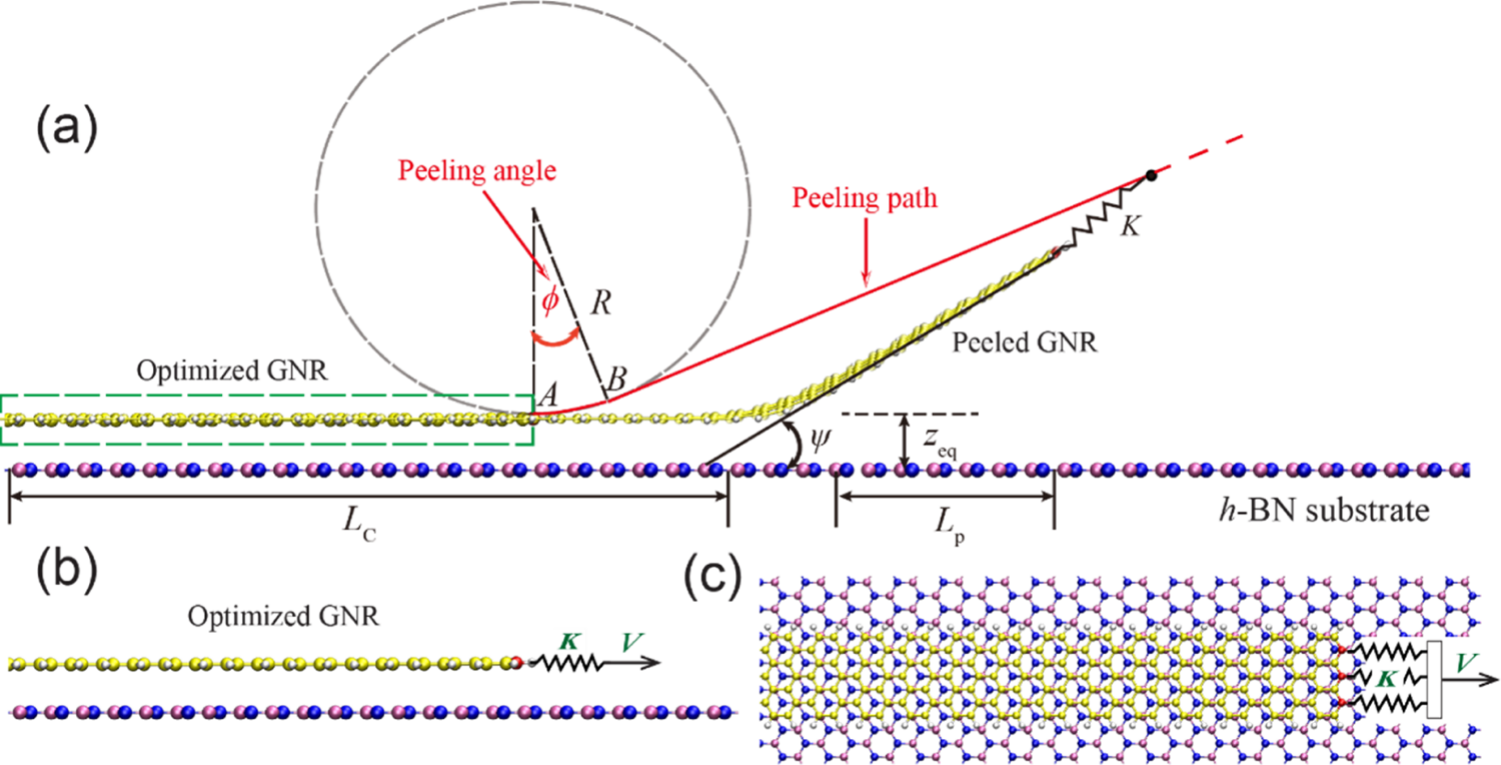
Schematic representation of the simulation setup. (a) GNR deposited over an h-BN substrate monolayer is peeled by a stage moving along the peeling path (red solid and dashed line). The stage is connected to the three rightmost carbon atoms (red spheres) of the GNR via springs of stiffness *K*. The peeling path is set by the peeling angle ϕ and the circle radius *R*. The angle between the peeled GNR section and the h-BN substrate is marked by ψ (see Section 3 of the Supporting Information). *L*_C_ and *L*_p_ are the contact length and the projected length of the peeled GNR on the substrate, respectively. Mauve, blue, yellow, and gray spheres represent boron, nitrogen, carbon, and hydrogen atoms, respectively. A section of the initially optimized GNR is illustrated within the green rectangle, with an equilibrium interlayer distance *z*_eq_. Side and top views of the relaxed GNR at the beginning of the peeling process are given in panels (b) and (c), respectively.

In the present work, we adopt the GNR/h-BN interface to investigate the process of quasi-one-dimensional materials peeling in both the superlubric and frictional regimes. We investigate the mechanisms of the detachment of an armchair GNR from an h-BN surface upon pulling of one end along different directions. Our model system consists of an armchair GNR of fixed width (∼0.7 nm) and length (∼20 nm) deposited on a rigid h-BN monolayer (see Figure 1). The GNR’s edges are passivated by hydrogen atoms^41^ to avoid peripheral C–C bond reconstruction,^47,48^ which may influence the friction and hence the peeling process. The GNR is initially placed atop the h-BN substrate in three different orientations aligning its long axis parallel to the (i) armchair (θ = 0°) and (ii) zigzag directions (θ = 90°) of the hexagonal surface, as well as (iii) 45° in between them (θ = 45°).

The construction of dedicated force fields for 2D materials and their layered stacks should account for their inherent structural anisotropy. Hence, a general strategy of combining force fields separately treating the intra- and interlayer interactions is widely adopted. Here, the intralayer C–C and C–H interactions within the GNR are evaluated via the reactive empirical bond order force field.^49^ The interlayer interactions between the GNR and the h-BN substrate are described by the dedicated registry-dependent interlayer potential (ILP)^50−53^ with refined parameters,^46^ which we implemented in the LAMMPS^54^ code. The validation of such a choice of force fields can be found in refs (53) and (55).

All simulations are performed adopting the following protocol. First, we generate the starting configurations of the GNR structures via geometry optimization using the FIRE algorithm,^56^ as implemented in LAMMPS,^54^ with a threshold force value of 10^–6^ eV/Å. Peeling simulations are then carried out by attaching the three rightmost carbon atoms of the GNR (red spheres in Figure 1), via springs of constant *K* = 3.33 N/m, to a stage of position ***r***^stage^(*t*) that is moving along the peeling path (see red solid and dashed lines in Figure 1). With this setup, the overall effective spring constant acting on the leading edge is 10 N/m, close to the typical values used in AFM experiments.^57^ The peeling path is divided into two parts: the stage first moves along the circumference of a circle of radius *R* = 1 nm from point *A* to point *B*, and after reaching a specific angle ϕ, it switches to unidirectional movement along the tangent line to the circle at point B. The angle ϕ is defined as the peeling angle.

**Figure 2:**
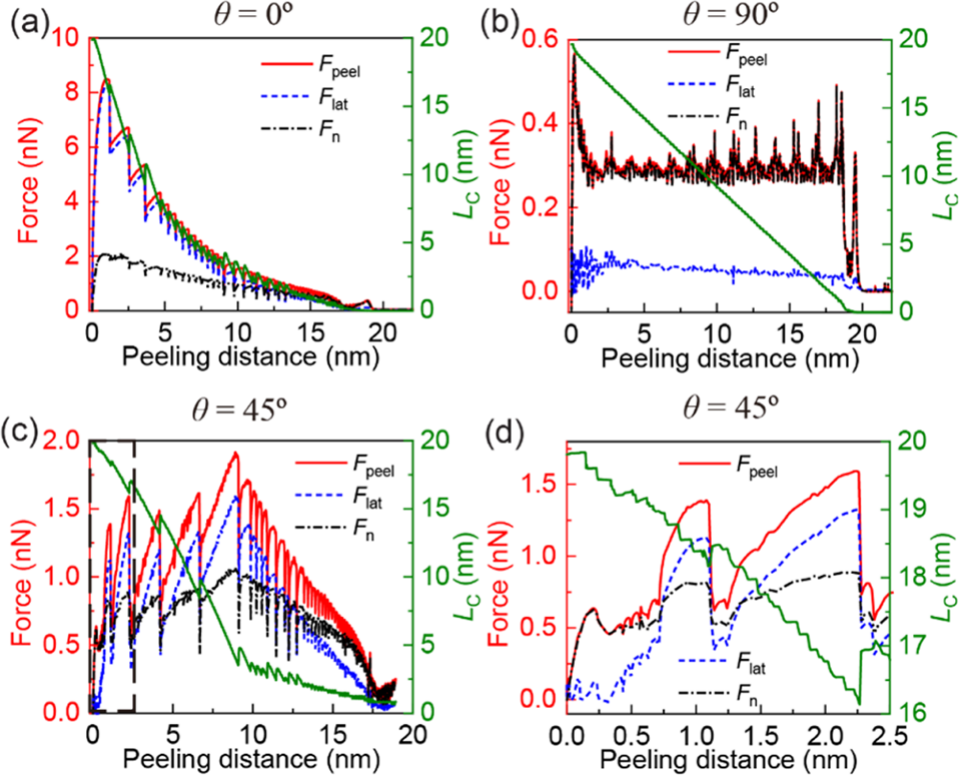
Quasi-static peeling of a 20 nm long GNR on a rigid h-BN substrate at a peeling angle of ϕ = 90°. Peeling force (red line), its lateral (dashed blue line) and normal (dashed-dotted black line) components (left axis) and contact length (right axis, green) as functions of peeling distance (defined as the length of the peeling path) for three different relative orientations of the GNR with respect to the h-BN substrate: (a) θ = 0°, (b) θ = 90°, and (c) θ = 45°. Panel (d) provides a zoom-in view of the initial 2.5 nm stage displacement during the peeling process, as marked by the dashed black rectangle in panel (c).

Quasi-static simulations are used to mimic low-speed experimental conditions. Within this procedure, at each step, the stage is shifted by 0.1 Å along the peeling path, then its position is fixed, and the whole system is allowed to relax using the FIRE algorithm^56^ with a force criterion of 10^–3^ eV/Å. Convergence tests using a tighter force criterion of 10^–6^ eV/Å provide similar results (see Section 1 of the Supporting Information). This process is repeated until the GNR fully detaches from the h-BN substrate. During the peeling process, the force applied to the leading edge of the ribbon is calculated as ***F***_peel_ = 3*K*(***R***_stage_ – ***R***_edge_), where ***R***_edge_ = ∑_*i*=1_^3^***R***_*i*,edge_/3 is the mean position of the GNR’s edge atoms. At the steady state, the trace average of |***F***_peel_| is defined as the peeling force and its maximal value is defined as the peel-off force. Here, the term steady state refers to regions of constant average force along the peeling trace (see Figure 2b).

To extract the adhesion energy from the peeling process, the contact length (*L*_C_) of the GNR during peeling should be monitored. To that end, the contact length is evaluated as follows: any atom in the GNR is considered to be in contact with the substrate if its vertical distance from the substrate’s surface is smaller than 4 Å, amounting for a binding energy of 40 meV/C atom (to be compared with 54.3 meV/C atom at the equilibrium interlayer distance of 3.3 Å). The GNR contact length is then given by the lateral distance along the long GNR axis between the tail atoms and the last atom, which is still in contact with the substrate. A sensitivity test of this approach toward the choice of the cutoff vertical distance is given in Section 2 of the Supporting Information.

## Results and Discussion

By applying this simulation protocol at various peeling angles to interfaces of different relative orientations between the GNR and the h-BN substrate, we reveal that the peeling process strongly depends on the latter. We start the discussion with the nearly commensurate (lattice mismatch of ∼1.8%) aligned (θ = 0°) contact, which exhibits a relatively large static friction (∼9.1 nN)^46^ (see Figures 2 and S5 in Section 4 of the Supporting Information). For this interface, when the peeling angle is in the range ϕ ≤ 90°, static friction resists the peeling process resulting in overall higher peeling forces, compared to other GNR orientations. In this case, peeling begins by a detachment of the leading edge of the GNR from the substrate, while the trailing GNR section remains immovable at contact with the substrate (see Figure 2a for a peeling angle of ϕ = 90°). The latter sticks to the surface until the force induced by the stage on the GNR exceeds the static friction force at the contacting section. At this point, a sudden slip event occurs (see Movie S1). For the 20 nm long GNR considered herein, as the stage advances along the peeling path, this stick-slip behavior repeats with reduced peak forces due to the reduction in the GNR contact length (and hence overall static friction force) up to full detachment. For small peeling angles (≲15° for the 20 nm long ribbon), the peak force initially remains constant until the contact length reduces below a threshold, where friction becomes length-dependent (see Figure S5 in Section 4 of the Supporting Information).^46^ Longer GNRs present a similar peeling behavior (see Figure S8 in Section 5 of the Supporting Information). When extracting the peak force before each slip event as a function of the corresponding contact length at various peeling angles for the 20 nm long GNR, an initial linear rise that levels off is observed (see Figure S11 in Section 6 of the Supporting Information). This indicates that the preslip stress distribution within the contacted GNR plays an important role in the peeling process for the aligned contact.^46^ We note that due to the strong size dependence of the interfacial friction, steady state is not achieved for the aligned contact during the entire peeling process. This breaks the steady-state assumption underlying some existing theoretical models.^29,58^ When the peeling angle, ϕ, is larger than 90°, a completely different behavior is found (see Figure S5d in Section 4 of the Supporting Information). Due to the high interfacial static friction, the GNR section in contact with the substrate, which is now being pushed and compressed (rather than pulled and stretched), does not slip. This results in pure detachment of the GNR from the substrate with no stick-slip motion (see Movies S2 and S3). Steady state can then be reached, and the corresponding peeling force depends on the peeling angle, such that when ϕ increases from 120 to 180°, the force decreases from 1.22 to 0.23 nN. We may therefore conclude that a larger peeling angle is advantageous when peeling interfaces of layered materials in the high static friction regime.

For the incommensurate θ = 90° interface, a superlubric contact is formed between the GNR and the underlying h-BN substrate. As a result, smooth-sliding peeling behavior is observed for peeling angles in the range ϕ ≤ 90° (see Movie S4) allowing for steady state to be reached (see Figure 2b for the vertical peeling, ϕ = 90°, case). This resembles peeling behaviors observed for other superlubric contacts, such as GNR/gold heterojunction,^44^ indicating the general nature of our treatment. Unlike the θ = 0° case, here, a similar behavior was also found for peeling angles of ϕ > 90° (see Figure S6 in Section 4 of the Supporting Information and Movie S5). The only exception found is for the extreme case of ϕ = 180°, where due to the superlow interfacial friction, the GNR was found to slide atop the h-BN substrate instead of being peeled-off (see Movie S6).

At an intermediate misfit angle of θ = 45°, the GNR peeling exhibits initial transient dynamics over a peeling distance of ∼1 nm followed by stick-slip peeling behavior (see Figures 2c,d and S7 in Section 4 of the Supporting Information). This is attributed to the reorientation of the GNR section in contact with the h-BN substrate during the peeling process yielding an effective θ = 60° partially commensurate contact (see Movies S7 and S8).^46^

**Figure 3:**
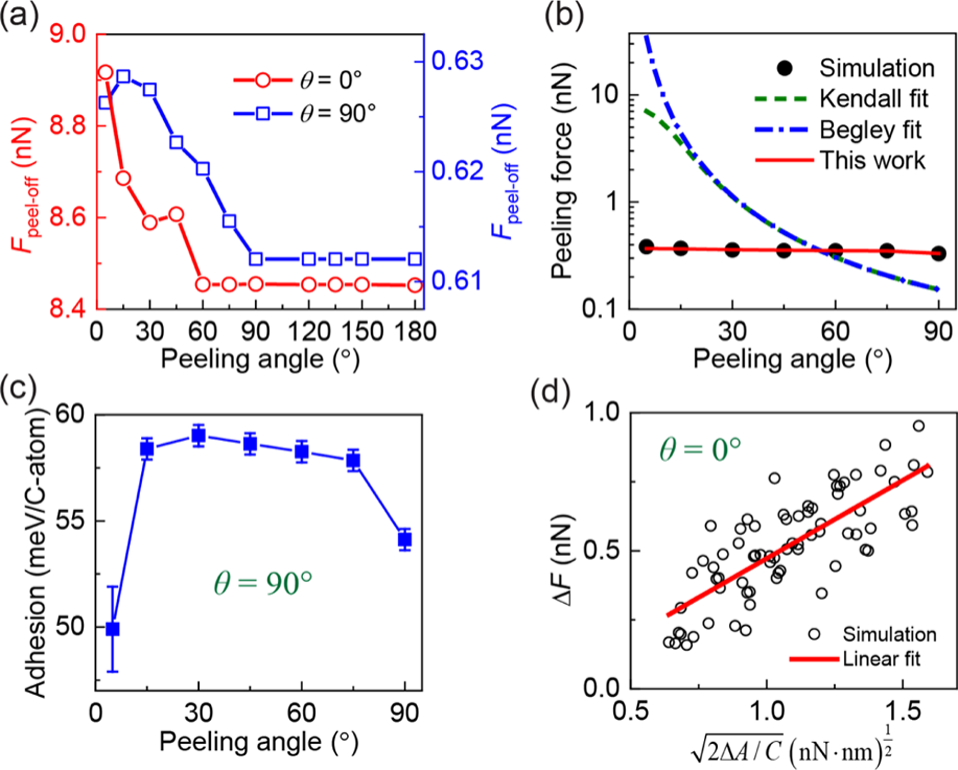
Dependence of the peeling process on the peeling angle. (a) Peel-off force at misfit angles of θ = 0° (open red circles, left *y*-axis) and θ = 90° (open blue squares, right *y*-axis). Note the different scales of the left and right *y*-axes. (b) Steady-state peeling force (full black circles) at a misfit angle of θ = 90° as a function of the peeling angle. The green, blue, and red curves in panel (b) are theoretical results calculated with eq 2 in ref (29) (see eq 1 herein), eq 31 in ref (58) (see eq 2 herein), and eq 4 herein, respectively. The value of the adhesion energy used in the equations is taken to be γ = 54.3 meV per carbon atom, which is the adhesion energy of graphene on h-BN calculated with the refined ILP.^46^ Panel (c) shows the estimated dependence of the adhesion energy (extracted from the peeling force traces via eq 4 for θ = 90°) on the peeling angle. In panel (d), the relation between the change in the peeling force, Δ*F*, and  for θ = 0° is shown for various peeling angles, where *C* and Δ*A* are the system’s compliance and the change of the contact area for each stick-slip event in the peeling force traces. The slope of the linear fit is 0.57 ± 0.05 (nN/nm)^1/2^, which corresponds to an adhesion energy of 52 ± 5 meV per carbon atom .

The analysis presented above indicates that apart from static friction, dictated by the interfacial commensurability, another quantity that may influence the peeling process is the peeling angle.^8,29,30,58^ To further investigate into this, we plot in Figure 3 the peel-off force and steady-state peeling force as a function of the peeling angle ϕ. We find that, regardless of the peeling angle, the peel-off force for the θ = 0° case is more than an order of magnitude larger than that obtained for θ = 90° (see Figure 3a), further demonstrating the important role of interfacial friction on the peeling process. For both misfit angles, the peel-off force exhibits a relatively weak dependence on the peeling angle with some reduction at low ϕ values and leveling-off at higher values (ϕ = 60° or 90° for θ = 0° or 90°, respectively).

Further important information regarding the contact adhesion can be extracted from the explicit dependence of the peeling force on the peeling angle. This, however, requires appropriate models providing a microscopic description of the peeling process in terms of the peeling procedure. A commonly used model for this purpose was developed by Kendall^29^ for steady-state peeling of an elastic film on a rigid substrate. Within this model, which assumes nonslip (pure stick) conditions, the steady-state peeling force, *F*_STS_, is related to the peeling angle via:
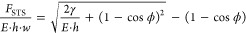
1where γ is the adhesion energy and *E*, *h*, and *w* are the Young’s modulus, the thickness, and the width of the elastic film, respectively. More recently, Begley et al.^58^ proposed an analytical model of the peeling of an elastic tape from a substrate, where large deformations of the tape and slip events within the adhered regions were considered. For single-side steady-state peeling and small strains, the critical force necessary to sustain peeling within this model is given by:
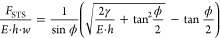
2

In Figure 3b, we compare the results of these two models (red and blue lines, respectively) to the steady-state peeling force obtained in our simulations (full black circles). To this end, the in-plane stiffness of the GNR is chosen as *E*·*h* = 26.6 eV/Å^2^,^59^ its width is taken to be the same as that used in the quasi-static simulations (*w* = 0.7 nm), and its adhesion energy with the h-BN substrate is chosen as γ = 54.3 meV per carbon atom as obtained from the refined ILP.^46^ The comparison clearly demonstrates that the two widely used steady-state peeling models fail to describe the peeling behavior of incommensurate GNR/h-BN contacts, where the steady-state peeling force is found to be independent of the peeling angle.

To explain this unexpected behavior, we developed an alternative model based on the method proposed by Lin and Zhao.^40^ In this model, at each quasi-static step, the vertical component of the external force, *F*_*z*_, acting on the driving end of the GNR, is balanced by the vdW interaction between the detached (*z* > *z*_eq_, see Figure 1) GNR section and the substrate. The GNR section that remains in contact with the surface at an equilibrium distance (*z* ≈ *z*_eq_) is assumed to experience no net vertical force. This translates to the following relation (see Section 7 of the Supporting Information)
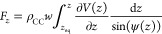
3where  is the number of carbon atoms per unit area, *a*_CC_ is the carbon–carbon bond length, *V*(*z*) is the vdW attraction potential between a carbon atom and the infinite flat substrate, and ψ(*z*) is the angle between the straight detached GNR section (see Section 3 of the Supporting Information) and the h-BN substrate (see Figure 1). Here, −∂*V*(*z*)/∂*z* is the vertical vdW force of an atom residing in the detached GNR section and separated by *z* > *z*_eq_ from the substrate and d*z*/sin(ψ(*z*)) is the differential length of this section. This quantity is integrated over the entire detached GNR segment, and the result is multiplied by the (constant) atom density in each such GNR section. The corresponding lateral forces in this case are assumed to be much smaller than *F*_*z*_ (see Figure 2b) and have minor contribution to the peeling force. With appropriate approximations (see Section 7 of the Supporting Information for further details), the vertical force as a function of height may be estimated as:
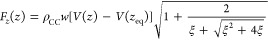
4where  and *D* is the bending rigidity of the GNR. In Figure S12 in Section 7 of the Supporting Information, we show that eq 4 fits well the simulation results for a misfit angle of θ = 90°, where the extracted binding energy *E*_bind_ (appearing in *V*(*z*)) is very close to the value calculated using the ILP and is independent of the peeling angle, as illustrated in Figure 3c.

The abovementioned model is not suitable for the case of the aligned (θ = 0°) GNR/h-BN contact because steady state is not reached in this case and the lateral force may have an important role in the peeling process. To extract the adhesion energy in this case, we adopt the following relation developed for reversible adhesive systems^9^ (see Section 8 of the Supporting Information for further details):
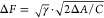
5where *C* is the compliance of the system (defined as the slope of the force trace during the stick stage), Δ*A* is the change of the contact area in each stick-slip event, and Δ*F* is the corresponding variation in the peeling force that can be extracted from the force traces (see Figure S13a in Section 8 of the Supporting Information). We note that along each force trace, there is a large variation of Δ*F*, Δ*A*, and *C* values that does not allow for a reliable estimation of γ from a single stick-slip event. Therefore, we plot in Figure 3d Δ*F* versus  for a large number of stick-slip events along force traces of different peeling angles for ϕ ≤ 90° (see Figure S5 in Section 4 of the Supporting Information) and extract γ from a linear fit to the scattered data. The obtained adhesion energy of  for the aligned GNR/h-BN heterojunction is in fair agreement with both the ILP value  and a recent experimental measurement .^60^

**Figure 4:**
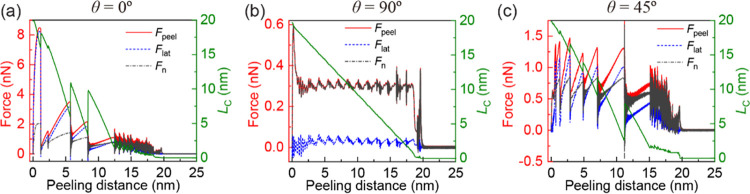
Constant velocity (*V* = 1 m/s) peeling of a 20 nm long GNR from an h-BN substrate. Presented are the peeling force (red line), its lateral (dashed blue line) and normal (dashed-dotted black line) components (left axis), and contact length (right axis, green) as functions of peeling distance (defined as the length of the peeling path) for three relative orientations of the GNR on the h-BN substrate: (a) θ = 0°, (b) θ = 90°, and (c) θ = 45°. The sharp transition appearing at a peeling distance of ∼11 nm for the θ = 45° case corresponds to a large slip event followed by readhering of part of the detached GNR section (see Movie S9).

**Figure 5:**
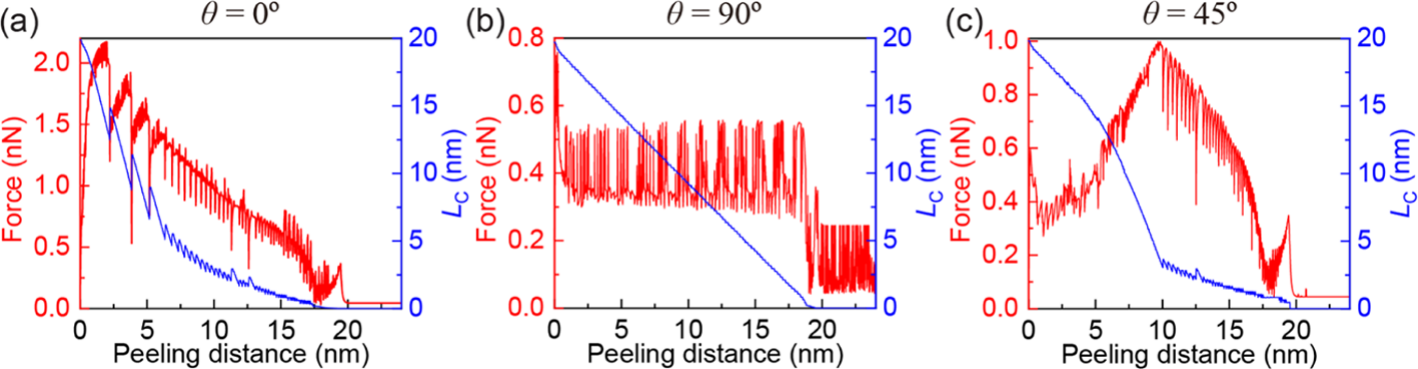
Quasi-static peeling of a 20 nm long GNR from an h-BN substrate calculated using the protocol of ref (44). The vertical peeling force (left axis) and contact length (right axis) as functions of peeling distance are presented for three different orientations of the GNR with respect to the h-BN substrate: (a) θ = 0°, (b) θ = 90°, and (c) θ = 45°.

In order to confirm the validity of our conclusions in the case of finite velocity peeling processes, we augmented the quasi-static peeling calculations with dynamical MD simulations. In what follows, we adopt a protocol in which the stage is shifted along the sliding path at a constant velocity of *V* = 1 m/s (see Supporting Information Section 5.2 and Figure 5 for similar results obtained using alternative peeling protocols^44,45^). The simulations are performed at zero temperature, where damped dynamics is applied to all GNR atoms with a damping coefficient of 0.5 ps^–1^. The simulation results for vertical peeling (ϕ = 90°) are illustrated in Figure 4 (peeling force traces for other peeling angles are provided in Figures S9 and S10 in Section 5 of the Supporting Information). The dynamic simulations support the quasi-static results, demonstrating peeling under stick-slip conditions for the aligned (θ = 0°) contact, smooth-sliding for the misaligned (θ = 90°) contact, and transient dynamics followed by stick-slip motion for the misaligned (θ = 45°) contact. The overall similar qualitative peeling behavior at the zero- and high-velocity limits indicates that the peeling mechanism in our setup is weakly dependent on velocity. The main differences are found for the aligned contact, where longer slip events accompanied by larger force drops are obtained at the high-velocity limit. We found that increasing the GNR length by 50% weakly affects the peeling behavior (see Section 5.2 of the Supporting Information), which is consistent with previous GNR friction results.^46^

The peeling protocol adopted herein, which includes elastic springs pulling the leading-edge atoms (see Figure 1), is not unique. To demonstrate that our general conclusions are independent of this choice, we repeated some of the peeling simulations using a different peeling protocol.^44,45^ Here, the in-plane lateral coordinates (*x*, *y*) of the leading-edge GNR atoms are kept fixed during the detachment process, and their vertical coordinate (*z*) is lifted quasi-statically without an explicit stage model or contacting springs (i.e., rigid contact). The results of these simulations are summarized in Figure 5. Like the protocol used in Figure 1, two distinct peeling regimes, i.e., stick-slip and smooth-sliding peeling, are observed for the aligned (θ = 0°) and misaligned (θ = 90°) contacts, respectively. For θ = 45°, the peeling behavior transits from smooth-sliding peeling to stick-slip peeling due to reorientation of the GNR with respect to the h-BN substrate during the peeling process. Thus, we can conclude that the qualitative peeling behavior of a GNR from the h-BN substrate is similar in both peeling protocols. We note, however, that quantitatively the magnitude of the peeling force for the aligned (θ = 0°) contacts is significantly smaller when using the rigid vertical shift protocol of ref (44). The reason is that restricting the lateral motion of the GNR leading-edge atoms during the peeling process reduces the effect of interfacial friction. The latter is expected to play a significant role in realistic peeling scenarios, where elastic effects between the ribbon and the pulling device are present. For misaligned contacts (θ = 90°), the value of the peeling force is similar in both protocols due to the ultralow friction force of the GNR in contact with the h-BN substrate.

## Conclusions

The results presented herein reveal an important aspect of the peeling of layered material interfaces, where the peeling mechanism is found to strongly depend on the relative orientation and hence the commensurability of the interface. When the lattice registry matching between the contacting surfaces is high, friction plays a central role in the peeling process resulting in a stick-slip regime of peeling and relatively high peeling forces. At low registry, a smooth peeling process is obtained, exhibiting a well-defined steady-state regime of lower peeling forces. For the case of GNR peeling from h-BN substrates, the peeling process seems to be weakly dependent on the peeling velocity and angle (up to ϕ = 90°, above which pure steady state peeling with no sliding is obtained, see Movies S2, S3, S5, and S8). Simple models were developed to extract the adhesion energy from the peeling force traces for both commensurate and incommensurate contacts. The microscopic understanding gained in this study and the suggested approaches for estimating the contact adhesion energy thus have important implications for controlling the exfoliation and transfer processes of layered materials involved in the design, fabrication, and applications of nanomechanical devices.

## Supporting Information Available

Convergence of the quasi-static simulations with respect to the force criterion; effect of the vertical distance criterion on the calculation of the contact length; the shape of the GNR during peeling; quasi-static peeling force traces with various peeling angles for a 20 nm long GNR; the peeling behavior for a 30 nm long GNR; the effect of the contact length on the maximum peeling force; theoretical model for extracting the adhesion energy from smooth-sliding peeling and from stick-slip peeling; and description of supplementary movies (PDF)
(MP4)
(MP4)
(MP4)
(MP4)
(MP4)
(MP4)
(MP4)
(MP4)
(MPG)

## Supplementary Material

am1c09529_si_009.mp4

am1c09529_si_010.mpg
