# Supplementary material for: Registry-Dependent Peeling of Layered Material Interfaces: The Case of Graphene Nanoribbons on Hexagonal Boron Nitride
Source: ACS Appl Mater Interfaces. 2021 Sep 5;13(36):43533–9. doi: 10.1021/acsami.1c09529 (PMC8488940; doi:10.1021/acsami.1c09529)
Supplement: Supplementary file 1 — am1c09529_si_001.pdf [file am1c09529_si_001.pdf]

Supplementary Information for “Registry Dependent Peeling of Layered Material Interfaces: the Case of Graphene Nanoribbons on Hexagonal Boron Nitride”

Wengen Ouyang[1], Oded Hod[2], and Michael Urbakh[2]*

[1]Department of Engineering Mechanics, School of Civil Engineering, Wuhan University, Wuhan, Hubei 430072, China

[2]Department of Physical Chemistry, School of Chemistry, The Raymond and Beverly Sackler Faculty of Exact Sciences and The Sackler Center for Computational Molecular and Materials Science, Tel Aviv University, Tel Aviv 6997801, Israel.

The supporting information includes the following sections:

1. Convergence of the quasi-static simulations with respect to the force criterion
2. Effect of vertical distance criterion on the calculation of the contact length
3. The shape of the GNR during peeling
4. Quasi-static peeling force traces with various peeling angles for a 20 nm long GNR
5. The peeling behavior for a 30 nm long GNR
6. The effect of contact length on the maximum peeling force
7. Theoretical model for extracting the adhesion energy from smooth-sliding peeling
8. Theoretical model for extracting the adhesion energy from stick-slip peeling
9. Description of the supplementary movies

Corresponding Author

*E-mail: odedhod@tauex.tau.ac.il.

1 Convergence of the quasi-static simulations with respect to the force criterion

All quasi-static simulations presented in the main text were performed using the FIRE algorithm[1] with a force criterion of 10^{-3} eV/Å. To verify the adequacy of this criterion we repeated some of the quasi-static peeling simulations of the aligned ($\theta = 0°$, **Figure S1**a) and misaligned ($\theta = 90°$, **Figure S1**b) contacts formed between a graphene nanoribbon (GNR) and a hexagonal boron nitride (*h*-BN) surface with a peeling angle of $\phi = 90°$ using a tighter force criterion of 10^{-6} eV/Å. As can be seen from **Figure S1**, both force criteria give similar peeling traces, indicating the convergence of the simulation results with respect to this parameter.

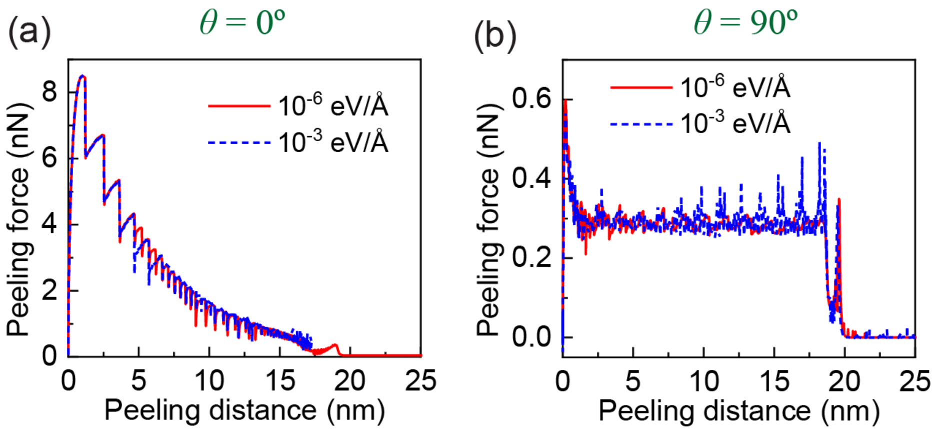

Figure S1. Convergence of the quasi-static peeling force trace with respect to the force criterion used in the FIRE algorithm[1] for two different relative orientations of the GNR and *h*-BN lattices: (a) $\theta = 0°$, and (b) $\theta = 90°$. A peeling angle of $\phi = 90°$ is used for both systems.

2 Effect of vertical distance criterion on the calculation of the contact length

As is common in atomic-scale simulations, the contact area between a surface and a substrate can be defined as the area covered by surface atoms that feel an overall repulsive force from the substrate. This corresponds to distances smaller than the equilibrium distance (z_{eq}).[2] In the GNR/*h*-BN system studied in this work, $z_{eq} = 3.3$ Å in the optimized configuration. To account for local fluctuations in the vertical positions of the atoms, we chose a somewhat larger value of $z_{cut} = 4$ Å $> z_{eq}$ as the criterion for the calculation of the contact length of the GNR. This allows us to obtain a reasonable measure of the contact area while avoiding sharp variations in its value due to atomic position fluctuations. To check the effect of the choice of z_{cut} on the calculated contact length (L_C), we lowered its value to 3.3 Å (corresponding to a binding energy of $\sim 54 \frac{\text{meV}}{\text{Carbon atom}}$) and doubled its value to 8 Å (with a binding energy of $\sim 1 \frac{\text{meV}}{\text{Carbon atom}}$) while recalculating the contact length using the same approach. The results of both calculations are compared in **Figure S2** with those obtain using the cutoff distance applied in the main text (4 Å). From this figure we can conclude that choosing a value of 3.3 Å results in pronounced fluctuations and that increasing z_{cut} to 8 Å induced only a slight shift in the estimated value of the contact length with no effect on its qualitative behavior during the entire simulated peeling process. This justifies the used of the value $z_{cut} = 4$ Å.

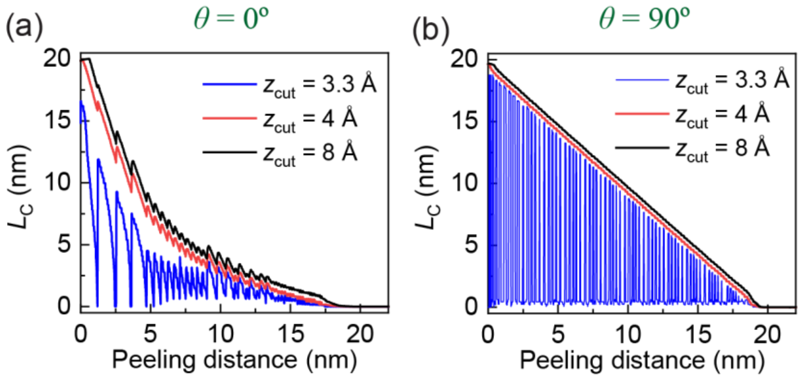

Figure S2. Effect of the choice of cutoff vertical distance on the calculated contact length for two different relative orientations of the GNR with respect to the *h*-BN substrate: (a) $\theta = 0°$, and (b) $\theta = 90°$. A peeling angle of $\phi = 90°$ is used for both systems.

3 The shape of the GNR during peeling

According to the model suggested by Gigli et al,[3] the shape of a GNR during peeling from a rigid substrate can be described by the following equation:

$$z = \begin{cases} z_{\text{eq}}, & x \in [0, L_C] \\ z_{\text{eq}} + R - \sqrt{R^2 - (x - L_C)^2}, & x \in [L_C, x_c], \\ z_c + (x - x_c)\tan(\psi)\,, & x \in [x_c, L] \end{cases} \tag{S1}$$

where $x_c = L_C + R\sin(\psi)$ and $z_c = z_{\text{eq}} + R[1 - \cos(\psi)]$. This equation describes the GNR as three connected sections: (i) a flat horizontal section in contact with the substrate, (ii) a circular section at the region of detachment, and (iii) a leading inclined straight section. To check the applicability of Eq. S1 to the studied GNR/h-BN systems, we extracted snapshots of the peeling process and fitted them to Eq. S1, the results are illustrated in **Figure S3**.

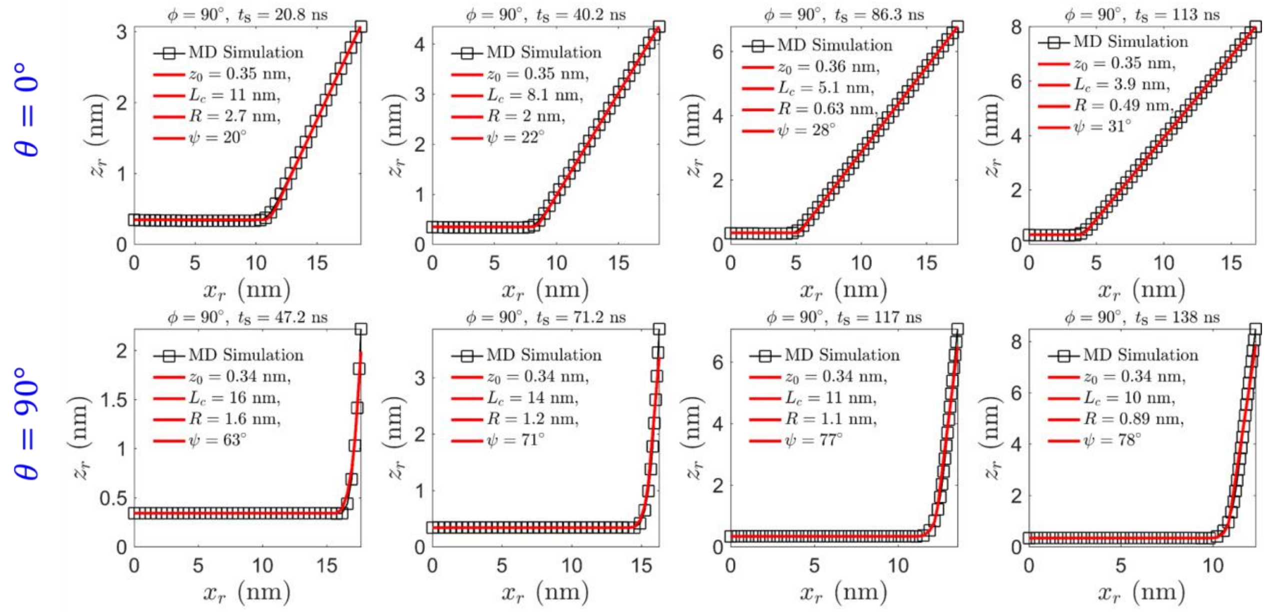

Figure S3. The shape of a 20 nm long GNR at various times (t_s) during quasi-static peeling simulations for the aligned ($\theta = 0°$, upper panels) and misaligned ($\theta = 90°$, lower panels) GNR/h-BN contacts, performed using the simulation protocol presented in the main text. Black squares and red lines correspond to the simulation data and fitting curves based on Eq. S1, respectively. The values of the fitting parameters are listed in each panel. The height of the GNR (z_r) is calculated by averaging the atomic height along the narrow dimension of the GNR (excluding the passivating hydrogen atoms) and over a single axial unit-cell. The position of each axial unit-cell along the GNR (x_r) is calculated as the distance of its center-of-mass from the ribbon's trailing edge.

Interestingly, although Eq. S1 is proposed only for superlubric contacts with smooth-sliding peeling behavior, this formula is found to be suitable also for the aligned GNR/h-BN contacts ($\theta = 0°$), where stick-slip peeling behavior is observed. The radius of curvature of the circular GNR section

is found to decrease during the peeling process. For the misaligned $(\theta = 90°)$ contact, the radius of curvature saturates at around 0.9 nm, which is slightly larger than the value reported by Gigli et al. $(\sim 0.8$ nm$)$ for a GNR peeled-off of a gold substrate.[3] We attribute this small difference to the different simulation protocols and substrates. To support this point, we performed additional simulations with the same protocol used in Ref. 4 (see Figure 5 in the main text). The corresponding GNR snapshots during the peeling process appear in **Figure S4**, which indeed shows even better agreement between the obtained saturated radius of curvature and that reported by Gigli et al.[3] for misaligned contacts $(\theta = 90°)$.

Overall, it should be noted that the exact shape of the GNR (and the corresponding elastic energy) during the peeling process has a minor effect on the obtained peeling forces.

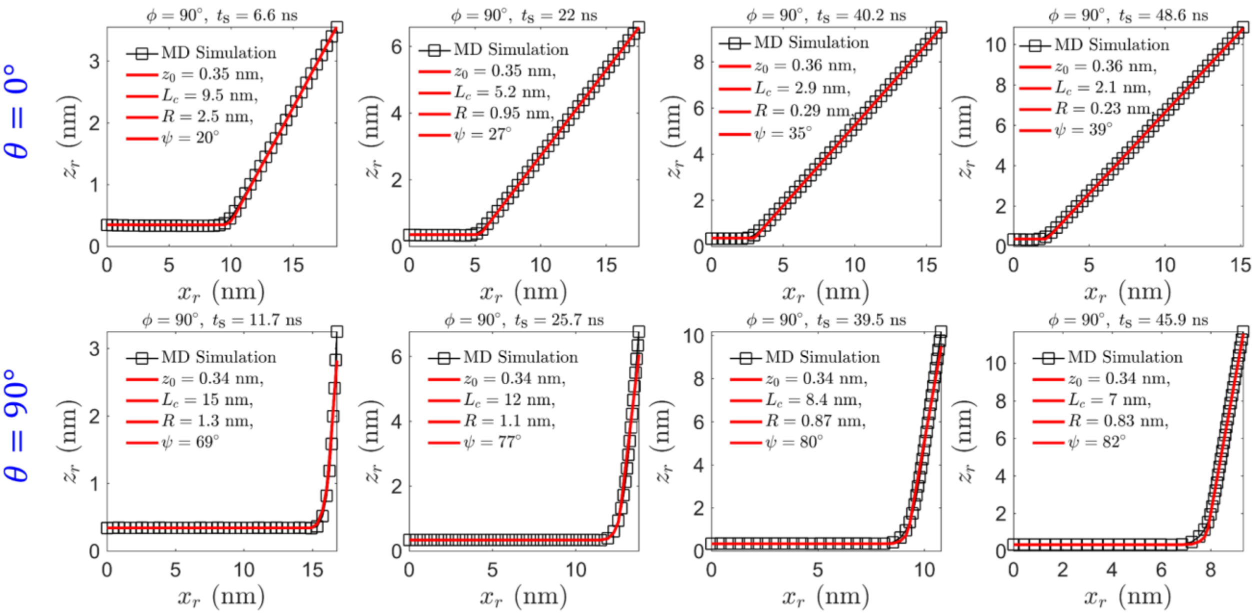

Figure S4. The shape of a 20 nm long GNR at various times (t_s) during quasi-static peeling simulation for the aligned ($\theta = 0°$, upper panels) and misaligned ($\theta = 90°$, lower panels) GNR/*h*-BN contacts performed using the simulation protocol of Ref. 4 (see Figure 5 in the main text). Black squares and red lines correspond to the simulation data and fitting curves based on Eq. S1, respectively. The values of the fitting parameters are listed in each panel. The height of the GNR (z_r) is calculated by averaging the atomic height along the narrow dimension of the GNR (excluding the passivating hydrogen atoms) and over a single axial unit-cell. The position of each axial unit-cell along the GNR (x_r) is calculated as the distance of its center-of-mass from the ribbon's trailing edge.

4 Quasi-static peeling force traces with various peeling angles for a 20 nm long GNR

In the main text, we presented peeling force traces of a GNR from a h-BN surface obtained at a peeling angle of $\phi = 90°$. For completeness, here we present complementary peeling force traces for several other peeling angles obtained using the quasi-static peeling protocol described in the main text. **Figure S5**-**Figure S7** show the peeling force (left axis) and the contact length (right axis) as a function of peeling distance along the peeling path with four peeling angles ($\phi = 5°,\ 30°,\ 75°$, and $135°$) and for three orientations ($\theta = 0°,\ 90°$, and $45°$) of the GNR with respect to the h-BN substrate.

Interestingly, for very low peeling angles (see **Figure S5**a) of the aligned contact $(\theta = 0°)$, the peeling force (red trace) is found to be initially independent of the peeling distance. This results from the fact that at large contact length, the static friction force is approaching a constant value.[5] At larger peeling angles, this effect is eliminated since already at the initial stages of the peeling process, the contact length considerably reduces resulting in a decrease of the static friction. For longer GNRs, the contact length during the early stages of the peeling process exceeds the threshold value below which the static friction becomes length dependent. Hence, a similar constant force regime is expected to be observed in this case also for larger peeling angle scenarios. Above a peeling angle of $\phi > 90°$ stick-slip peeling is completely eliminated. Instead, pure peeling with no interfacial sliding motion is obtained due to the high interfacial static friction (see Supplementary Movies 2-3).

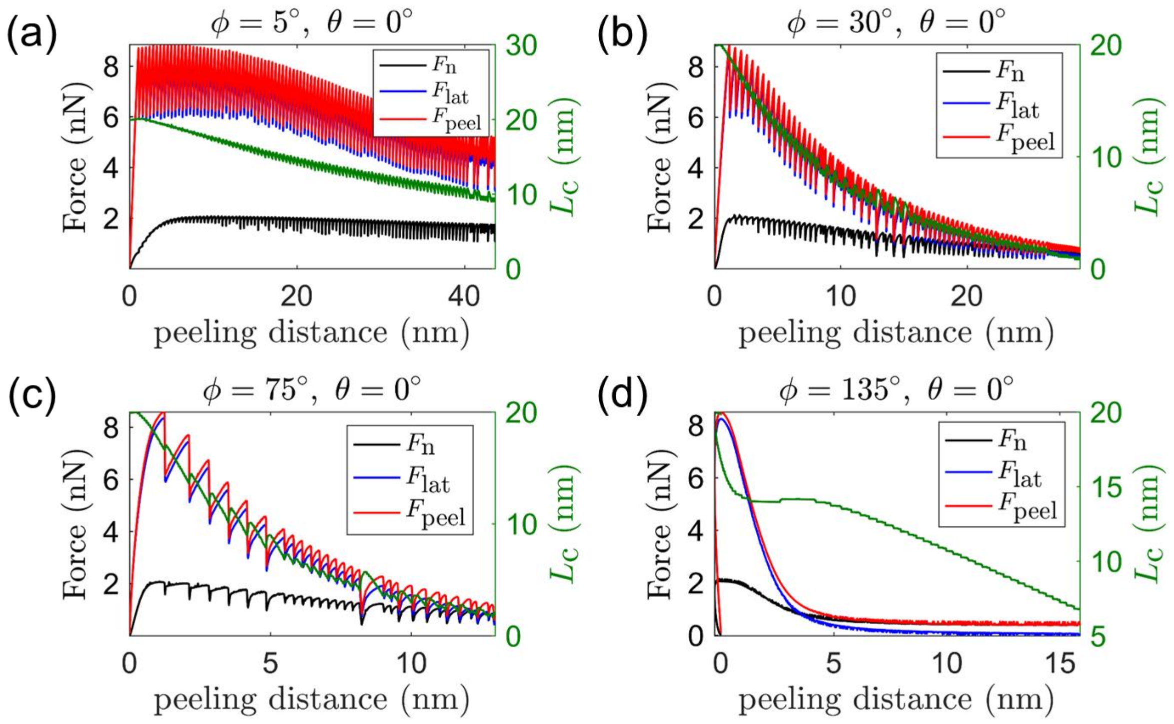

Figure S5. Quasi-static peeling of the aligned $(\theta = 0°)$ interface between a 20 nm long GNR and an *h*-BN substrate. Peeling forces (left axis) and contact length (right axis) as functions of the peeling distance for various peeling angles: (a) ϕ = 5°, (b) ϕ = 30°, (c) ϕ = 75°, and (d) ϕ = 135°.

For the misaligned ($\theta = 90°$, **Figure S6**) contact the overall peeling forces are found to be an order of magnitude smaller than those of the aligned contact. After initial transient dynamics a steady-state, characterized by force oscillations, is obtained for all peeling angles considered.

At a misfit angle of $\theta = 45°$ (see **Figure S7**) a more complex behavior is obtained involving intermediate peeling forces with pronounced oscillations and complex stick-slip motion, for peeling angles below $\phi = 90°$. This is associated with the reported reorientation of the GNR during the mixed peeling and sliding process.

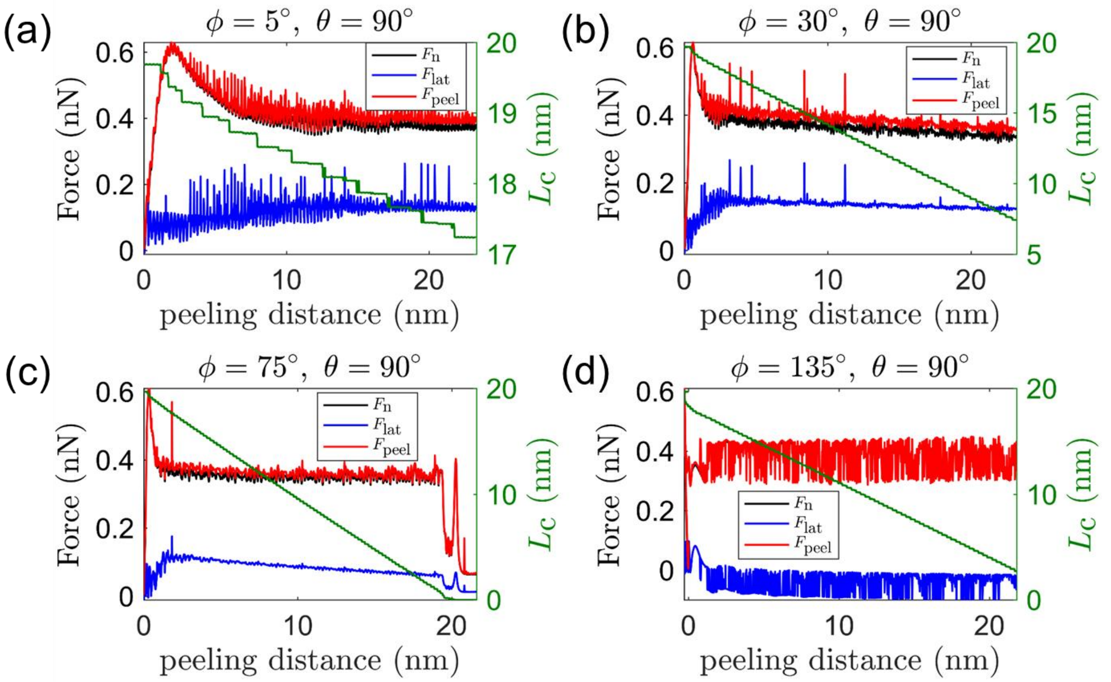

Figure S6. Quasi-static peeling of the misaligned $(\theta = 90°)$ interface between a 20 nm long GNR and an *h*-BN substrate. Peeling forces (left axis) and contact length (right axis) as functions of the peeling distance for various peeling angles: (a) ϕ = 5°, (b) ϕ = 30°, (c) ϕ = 75°, and (d) ϕ = 135°.

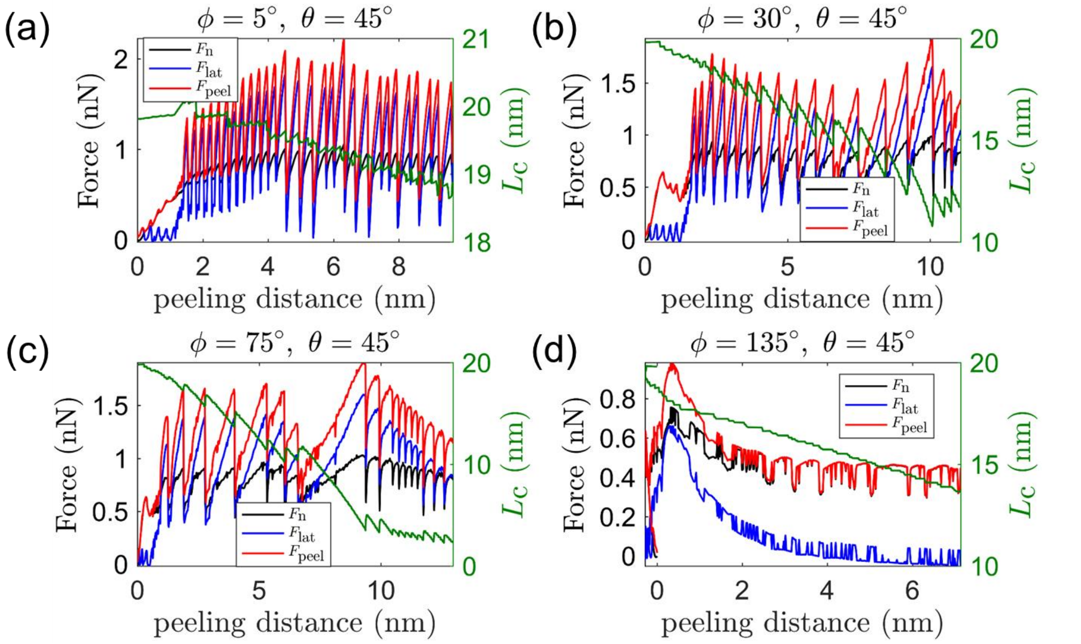

Figure S7. Quasi-static peeling of the misaligned $(\theta = 45°)$ interface between a 20 nm long GNR and an *h*-BN substrate. Peeling forces (left axis) and contact length (right axis) as functions of the peeling distance for various peeling angles: (a) ϕ = 5°, (b) ϕ = 30°, (c) ϕ = 75°, and (d) ϕ = 135°.

5 The peeling behavior for a 30 nm long GNR

5.1 *Quasi-static peeling*

In the main text, all peeling simulations were performed with a 20 nm long GNR. To check the effect of the GNR's length on its peeling behavior, we performed additional quasi-static simulations for a 30 nm long GNR using the FIRE algorithm[1] with a force criterion of 10^{-6} eV/Å. Both aligned ($\theta = 0°$) and misaligned ($\theta = 90°$) GNR/*h*-BN contacts were considered with two peeling angles of $\phi = 60°$ and 90°. The peeling force traces and the corresponding evolution of the contact length are presented in **Figure S8**, which shows a qualitatively similar peeling behavior as that for a 20 nm long GNR.

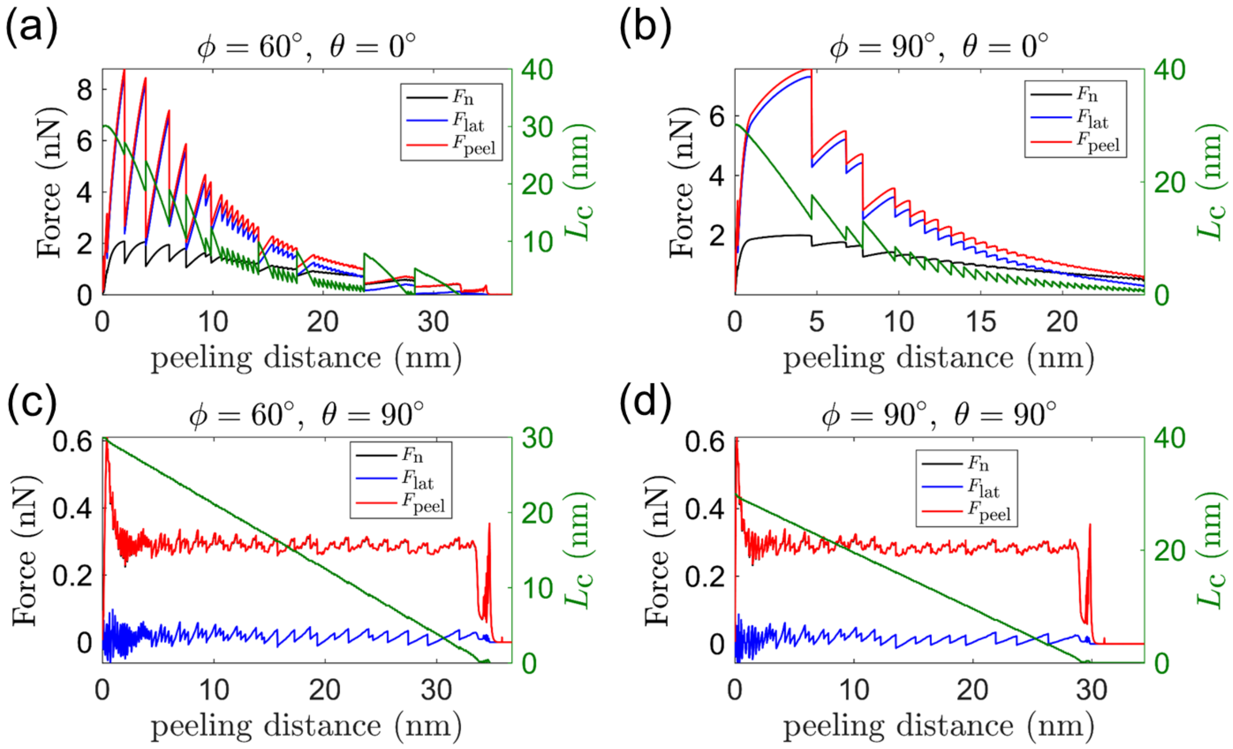

Figure S8. Quasi-static peeling of a 30 nm long GNR atop a *h*-BN substrate. Peeling forces (left axis) and contact length (right axis) as functions of the peeling distance for various orientation and peeling angles: (a) $\theta = 0°$, $\phi = 60°$, (b) $\theta = 0°$, $\phi = 90°$, (c) $\theta = 90°$, $\phi = 60°$ and (d) $\theta = 90°$, $\phi = 90°$.

5.2 *Constant velocity peeling*

In this section, we present representative peeling force traces of a 30 nm long GNR on an *h*-BN substrate, obtained using the constant velocity peeling protocol with a driving velocity of 1 m/s, for various peeling angles. **Figure S9** and **Figure S10** show the peeling force (left axis) and the contact length (right axis) as a function of peeling distance along the peeling path for four peeling angles ($\phi = 5°$, 30°, 90°, and 135°) and two orientations ($\theta = 0°$ and 90°) of the GNR with respect to the *h*-BN substrate. Comparing to the quasi-static results presented above, we find that the

qualitative behavior of the peeling force and contact length, obtained using the two protocols, is similar. The magnitudes of the peeling forces are also similar with some differences in the amplitude of the force and the contact area oscillations.

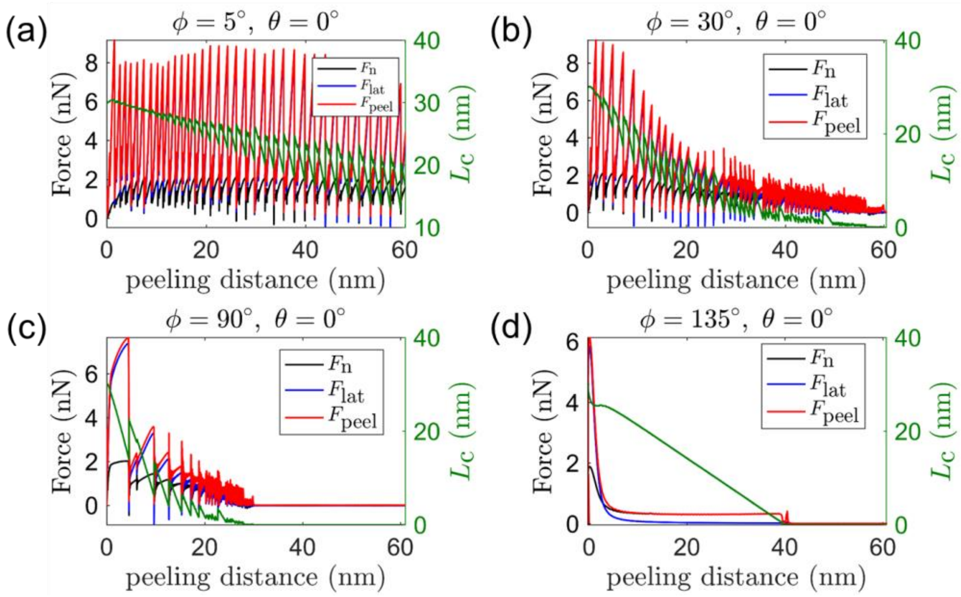

Figure S9. Constant velocity (1 m/s) peeling of the aligned ($\theta = 0°$) interface between a 30 nm long GNR and an *h*-BN substrate. Peeling forces (left axis) and contact length (right axis) as functions of the peeling distance for various peeling angles: (a) ϕ = 5°, (b) ϕ = 30°, (c) ϕ = 90° and (d) ϕ = 135°.

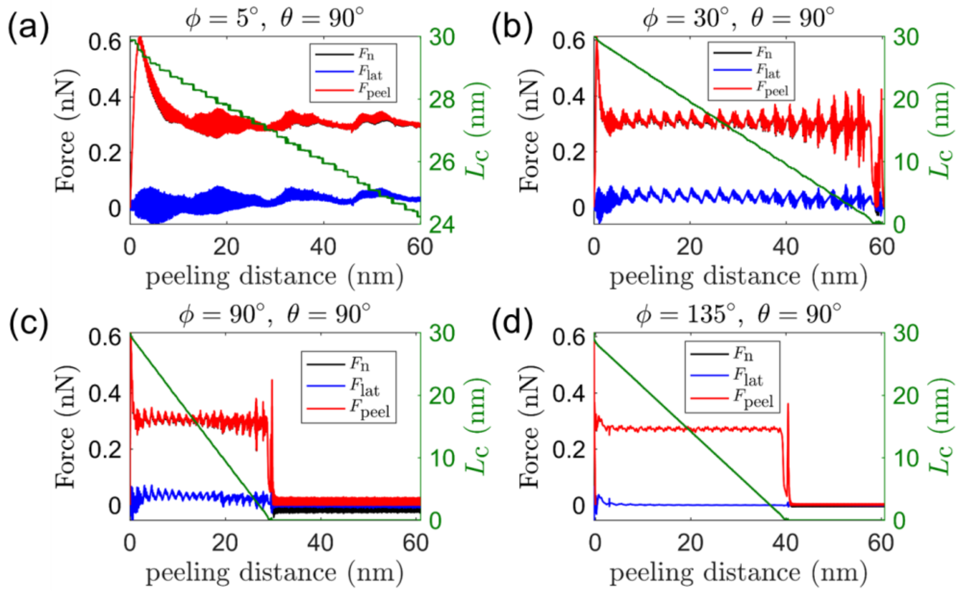

Figure S10. Constant velocity (1 m/s) peeling of the misaligned ($\theta = 90°$) interface between a 30 nm long GNR and an *h*-BN substrate. Peeling forces (left axis) and contact length (right axis) as functions of the peeling distance for various peeling angles: (a) ϕ = 5°, (b) ϕ = 30°, (c) ϕ = 90° and (d) ϕ = 135°.

6 The effect of contact length on the maximal peeling force

In Figure 2 of the main text, we found that the peeling force traces for the aligned GNR/h-BN contact $(\theta = 0°)$ show stick-slip behavior with a continuous overall reduction of the peeling force during the detachment of the GNR from the h-BN substrate with no apparent steady state. We attributed this behavior to the contact length dependence of the interfacial friction (and hence the peeling force) in the aligned GNR/h-BN contact, as presented in our recent study.[5] To further demonstrate this, we plot in **Figure S11**, the maximal peeling force as a function of contact length throughout the stick-slip traces of the aligned contact $(\theta = 0°)$ formed between a $20\ \text{nm}$ long GNR and an h-BN substrate. For all peeling angles considered in the range $\phi \leq 30°$, the maximal peeling force is constant at large contact lengths $(\gtrsim 15\ \text{nm})$ and depends linearly on the length at shorter contacts. For larger peeling angles of the $20\ \text{nm}$ long GNR, we are able to probe only the linear dependence on contact length regime. Simulations with longer ribbons are thus required to obtain the plateau region. The fact that the same contact length dependence of the maximal peeling force is obtained for all peeling angles indicates that this behavior originates from the interfacial friction.

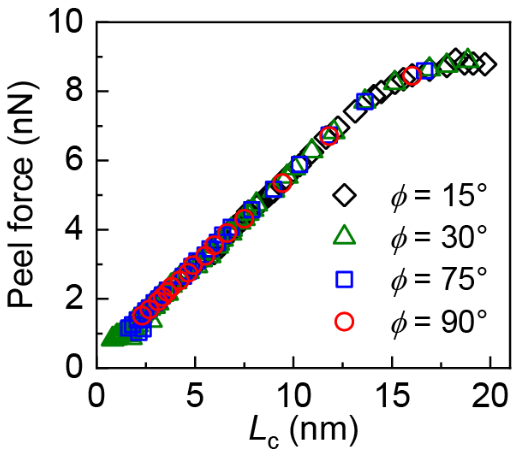

Figure S11. The maximal peeling force as a function of contact length for the aligned $(\theta = 0°)$ GNR/h-BN contact.

7 Theoretical model for extracting the adhesion energy from smooth-sliding peeling

In the main text, we showed that existing steady-state peeling models fail to describe the peeling behavior of incommensurate GNR/h-BN contact, where the steady-state peeling force is independent of the peeling angle. To describe this unexpected behavior, we develop a simple model using the method proposed by Lin et al.[6] In this model, the vertical force acting on the driving end of the GNR at steady-state is balanced by the vdW interaction between the substrate and the detached section of the GNR, which is defined by a vertical separation exceeding the equilibrium distance, z_{eq}. The remaining GNR section in contact with the substrate is assumed to reside at the equilibrium distance and hence does not contribute to the vertical force. Under these assumptions, the vertical force can be written as follows:

$$F_{\mathrm{z}} = \rho_{\mathrm{CC}} w \int_{z_{eq}}^{z} \frac{\partial V(z)}{\partial z} \frac{\mathrm{d}z}{\sin(\psi(z))}, \tag{S2}$$

where $\rho_{\mathrm{CC}} = \frac{4\sqrt{3}}{9a_{\mathrm{CC}}^2}$ is the number of carbon atoms per unit area, a_{CC} is the carbon-carbon bond length, w is the width of the GNR, $V(z)$ is the vdW interaction between a carbon atom and the infinite substrate, and $\psi(z)$ is the angle between the tangent of the GNR and the positive direction of the long axis of the GNR (see Figure 1 in the main text). Here, $-\frac{\partial V(z)}{\partial z}$ is the vertical vdW force of an atom residing in the detached GNR section and separated by $z > z_{eq}$ from the substrate and $\mathrm{d}z/\sin(\psi(z))$ is the differential length of this section. This quantity is integrated over the entire detached GNR segment and the result is multiplied by the (constant) atom density in each such GNR section.

To proceed, one needs to provide an explicit expression for the potential $V(z)$. In our numerical simulations we used the dedicated interlayer potential (ILP)[7-10] as detailed in the main text. Since this potential does not allow for an analytical solution of Eq. S2 and since this equation considers large separations between the peeled GNR section and the h-BN substrate, it is sufficient to use a Lennard-Jones (LJ) type potential to describe the long-range interaction appearing in Eq. S2. Adopting the standard 6-12 LJ potential functional form, the interaction between a carbon atom and an infinite flat substrate takes the following form[11]:

$$V(z) = 8\pi\rho_{\mathrm{BN}}\epsilon\sigma^2 \left[\frac{1}{10}\left(\frac{\sigma}{z}\right)^{10} - \frac{1}{4}\left(\frac{\sigma}{z}\right)^{4}\right], \tag{S3}$$

where $\rho_{\mathrm{BN}} = 4\sqrt{3}/9a_{\mathrm{BN}}^2$ is the number of B and N atoms per unit area and a_{BN} is the boron-nitrogen bond length. The parameters ϵ and σ are functions of the binding energy, E_{b}, and the equilibrium distance, z_{eq}, of the GNR on h-BN with the following form:

$$\epsilon = \frac{5E_\mathrm{b}}{6\pi\rho_\mathrm{BN} z_\mathrm{eq}^2}, \sigma = z_\mathrm{eq}, \tag{S4}$$

Substituting Eq. S4 into Eq. S3, $V(z)$ can be written as:

$$V(z) = \frac{20E_\mathrm{b}}{3}\left[\frac{1}{10}\left(\frac{z_\mathrm{eq}}{z}\right)^{10} - \frac{1}{4}\left(\frac{z_\mathrm{eq}}{z}\right)^{4}\right]. \tag{S5}$$

In the peeled region, the repulsive energy is negligible, thus we can approximate $V(z)$ as:

$$V(z) \approx -\alpha\frac{5E_\mathrm{b}}{3}\left(\frac{z_\mathrm{eq}}{z}\right)^{4}, \tag{S6}$$

where the parameter α mimics the effect of taper function and the short-range Fermi−Dirac type damping function in the attractive long-range interaction term of the ILP,[9] which are replaced here by the LJ potential.

To further simplify Eq. S2, we assume that $\psi(z)$ is approximately constant (see Section 3 above) and given by the angle at the middle of the peeled region, which is denoted by ψ_m. With this, Eq. S2 can be integrated analytically, yielding:

$$F_\mathrm{z} = \frac{\rho_\mathrm{CC} w}{\sin(\psi_\mathrm{m})}\left[V(z) - V(z_\mathrm{eq})\right], \tag{S7}$$

To obtain an expression for $\sin(\psi_\mathrm{m})$, we use continuum elastic beam theory, where the radius of curvature, R, of a beam under the action of a moment, M, is given by $R = Dw/M$, where D is the bending rigidity the beam. Using the expression for the radius of curvature of a general one-dimensional function $z(x)$: $1/R = z''/(1+z'^2)^{3/2}$, where $z' = \frac{\mathrm{d}z}{\mathrm{d}x} = \tan(\psi)$, we have:

$$\frac{\mathrm{d}\tan(\psi)}{[1+\tan^2(\psi)]^{3/2}} = \frac{M(x)}{Dw}\mathrm{d}x = \frac{F_z(L_p - x)}{Dw}\mathrm{d}x, \tag{S8}$$

where L_p is the projected length of the peeled section (see Figure 1 in the main text) and the origin is defined as the position of the trailing edge of the GNR. Integrating both sides of Eq. S8 yields:

$$\sin\psi = \frac{F_z}{Dw}\left(L_p x - \frac{x^2}{2}\right). \tag{S9}$$

Setting $x = L_p/2$, we then have:

$$\sin\psi_\mathrm{m} = \frac{3F_z L_p^2}{8Dw}. \tag{S10}$$

Substituting Eq. S10 into Eq. S7, we have:

$$\sin^2(\psi_\mathrm{m}) = \frac{3\rho_\mathrm{CC} L_p^2}{8D}\left[V(z) - V(z_\mathrm{eq})\right]. \tag{S11}$$

From the simulation results, we found that the peeled GNR section is nearly straight, thus the projected length, L_p, can be approximated as:

$$L_p = (z - z_\mathrm{eq})/\tan(\psi_\mathrm{m}). \tag{S12}$$

Substituting Eq. S12 into Eq. S11 and using the trigonometric relation $\sin^2(\psi_\mathrm{m}) = \frac{\tan^2(\psi_\mathrm{m})}{1+\tan^2(\psi_\mathrm{m})}$, we

have:

$$\frac{\tan^4(\psi_\mathrm{m})}{1+\tan^2(\psi_\mathrm{m})} = \frac{3\rho_\mathrm{CC}(z-z_\mathrm{eq})^2}{8D}\left[V(z)-V(z_\mathrm{eq})\right] \equiv \xi. \tag{S13}$$

The solution of Eq. S13 is:

$$\tan^2(\psi_\mathrm{m}) = \left(\xi + \sqrt{\xi^2+4\xi}\right)/2. \tag{S14}$$

Substituting Eq. S14 in Eq. S7 and using the same trigonometric relation as above, the vertical force as a function of height z can be expressed as:

$$F_\mathrm{z} = \rho_\mathrm{CC} w\left[V(z)-V(z_\mathrm{eq})\right]\sqrt{1+\frac{2}{\xi+\sqrt{\xi^2+4\xi}}} = \frac{8Dw\xi}{3(z-z_\mathrm{eq})^2}\sqrt{1+\frac{2}{\xi+\sqrt{\xi^2+4\xi}}}. \tag{S15}$$

Figure S12 compares results obtained using the above model with the simulation results for the misaligned $(\theta = 90°)$ contact, which exhibits smooth-sliding peeling. Eq. S15 is fitted to the simulations results (red lines in **Figure S12**) using a single parameter, namely the binding energy, E_bind, appearing in Eq. S5. The equilibrium distance z_eq and the constant α are fixed at 3.3 Å and 0.8, respectively. A very good agreement between the simulation results and the model predictions is obtained, where the fitted binding energies agree well with the value calculated from the ILP (54.3 meV/C atom). We note that using $\alpha = 1.0$ results in binding energies that are ~10% lower than that predicted by the ILP. Importantly, the peeling force predicted by Eq. S15 is found to be weakly dependent on the peeling angle for the misaligned $(\theta = 90°)$ contact.

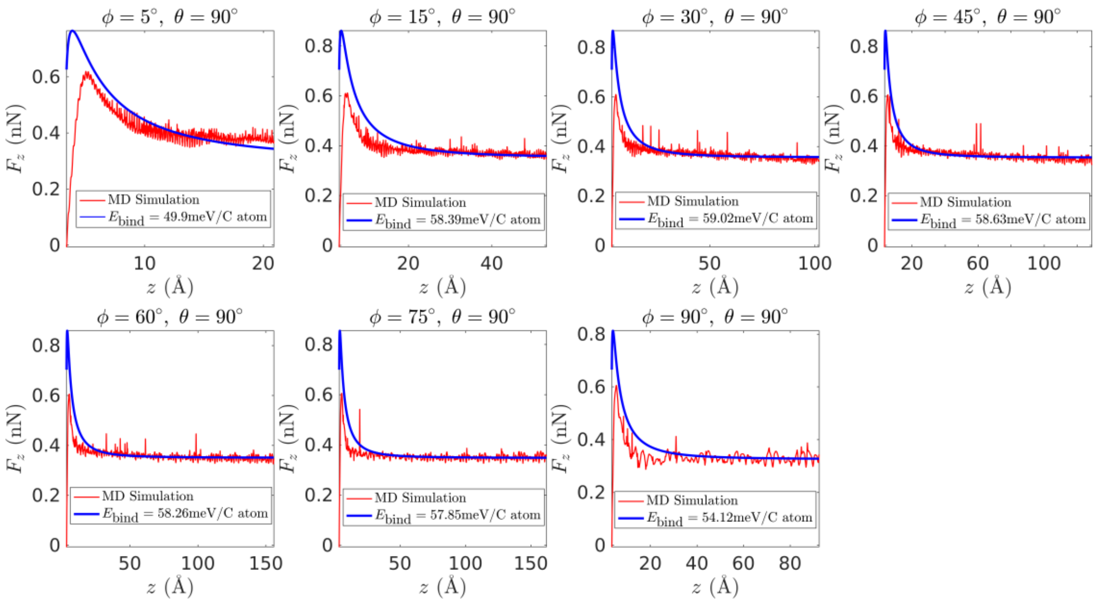

Figure S12. Normal peeling force (F_z) as a function of vertical peeling distance (z) for the misaligned $(\theta = 90°)$ contact between a 20 nm long GNR and an h-BN substrate at various peeling angles (ϕ). The red and blue lines represent simulation and model results (Eq. S15), respectively.

8 Theoretical model for extracting the adhesion energy from stick-slip peeling

As seen in our simulations, in the case of stick-slip peeling, steady-state is not achieved. Therefore, the model presented in section 7 above, is not suitable to treat this case. Therefore, in order to extract the adhesion energy from the stick-slip peeling force traces observed for the aligned ($\theta = 0°$) GNR/h-BN contact, we rely on energy conservation considerations rather than force balancing.[12, 13] Here, the variation of the adhesion energy between two consecutive stick events is given by:

$$\Delta E_{\mathrm{adh}} = (\gamma_1 + \gamma_2)\Delta A - \gamma_{12}\Delta A = \gamma\Delta A, \tag{S16}$$

where γ_1, γ_2 and γ_{12} are the surface energy densities of the GNR, the h-BN substrate, and the GNR/h-BN contact, respectively, $\gamma \equiv \gamma_1 + \gamma_2 - \gamma_{12}$, $\Delta A = \Delta L_c w$, and ΔL_c is the change in contact length between the two stick events (see **Figure S13**a). Assuming a dissipationless process, this energy change should be balanced by the sum of changes in the elastic energy stored in the GNR, ΔU_{el}, and the external work performed by the peeling force, $\delta\mathrm{W}$:

$$\Delta E_{\mathrm{adh}} = \Delta U_{\mathrm{el}} + \delta\mathrm{W}. \tag{S17}$$

According to our simulations ΔU_{el} is smaller than $\delta\mathrm{W}$ (see **Figure S13**b). Therefore, for simplicity we discard it in Eq. S17, yielding:

$$\gamma = \delta\mathrm{W}/\Delta L_c w. \tag{S18}$$

In the linear regime, where the peeling force depends almost linearly on the stage displacement, the work during each stick phase can be approximated as:

$$\delta\mathrm{W} = \frac{1}{2}C(\Delta F)^2, \tag{S19}$$

where C and ΔF are the compliance of the system and the change in the peeling force, respectively. Therefore, the adhesion energy density in each stick step is given by:

$$\gamma = \frac{1}{2}\frac{C(\Delta F)^2}{\Delta L_c w}. \tag{S20}$$

Eq. S20 allows for calculating the adhesion energy density directly from the peeling force traces. Due to variance between different stick events, a more reliable estimation may be obtained by averaging the result of Eq. S20 over many stick events along the peeling force trace. **Figure S13**c shows the calculated averages and the corresponding error bars extracted from the variance.

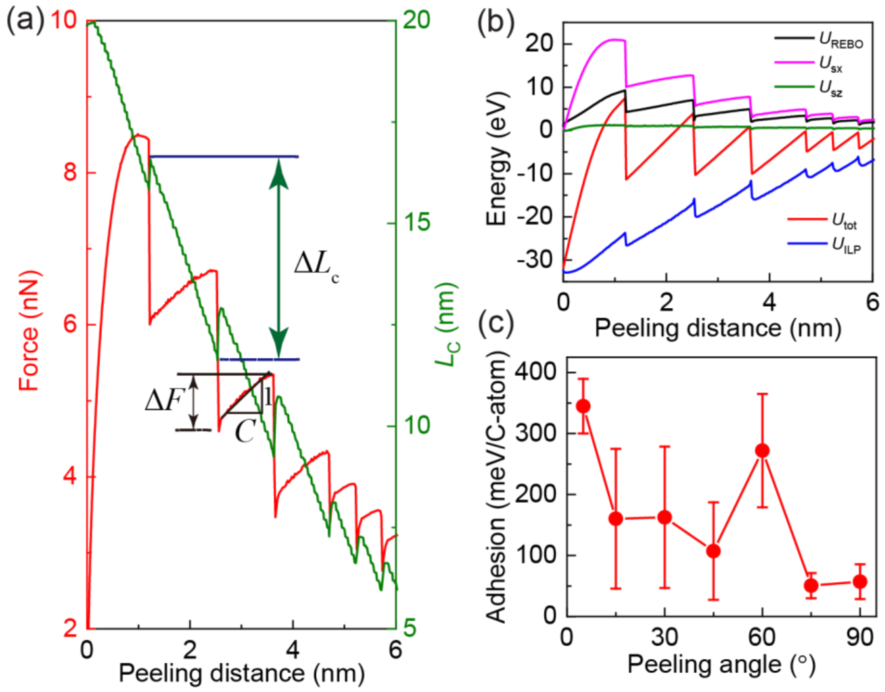

Figure S13. Extraction of the adhesion energy of the aligned GNR/*h*-BN contact $(\theta = 0°)$ under stick-slip conditions. Panels (a) and (b) correspond to the peeling force (red) and energies as a function of peeling distance at a peeling angle of $\phi = 90°$, respectively. Panel (a) also shows the contact length (right vertical axis, green) as a function of peeling distance. The parameters labeled in panel (a), i.e., C, ΔF and ΔL_c correspond to the system compliance, the change in the peeling force, and the change in the contact length, respectively, during a given stick event. Panel (b) shows the profiles for the total potential energy (U_{tot}, red line), the interlayer energy (U_{ILP}, blue line), the intralayer (elastic) energy (U_{REBO}, black line), and the work of applied force (stored in the driving springs) along the lateral pulling, *x,* (U_{sx}, green line) and vertical, *z,* (U_{sz}, orange line) directions. Zero energy corresponds to the state where the GNR is fully detached from the *h*-BN substrate. Panel (c) shows the estimated adhesion energy as a function of peeling angle using Eq. S20.

Eq. S20 can be rewritten as:[13]

$$\Delta F = \sqrt{\gamma} \cdot \sqrt{2\Delta A/C}, \tag{S21}$$

showing that the adhesion energy can be extracted by recording ΔF values as a function of $\sqrt{2\Delta A/C}$ from many peeling traces of various peeling angles, as illustrated in Figure 3 in the main text. A linear fit of this data gives a reasonable estimation of the adhesion energy for the GNR/*h*-BN heterojunction.

9 Description of the supplementary movies

GNR peeling trajectories obtained using the quasi-static simulation protocol:

Movie S1: $\theta = 0°$ and $\phi = 90°$ GNR peeling trajectory;

Movie S2: $\theta = 0°$ and $\phi = 135°$ GNR peeling trajectory;

Movie S3: $\theta = 0°$ and $\phi = 180°$ GNR peeling trajectory;

Movie S4: $\theta = 90°$ and $\phi = 90°$ GNR peeling trajectory;

Movie S5: $\theta = 90°$ and $\phi = 135°$ GNR peeling trajectory;

Movie S6: $\theta = 90°$ and $\phi = 180°$ GNR peeling trajectory;

Movie S7: $\theta = 45°$ and $\phi = 90°$ GNR peeling trajectory;

Movie S8: $\theta = 45°$ and $\phi = 180°$ GNR peeling trajectory;

GNR peeling trajectories obtained using the constant velocity simulation protocol:

Movie S9: $\theta = 45°$ and $\phi = 90°$ GNR peeling trajectory.

References

(1) Bitzek, E.; Koskinen, P.; Gähler, F.; Moseler, M.; Gumbsch, P. Structural Relaxation Made Simple. *Phys. Rev. Lett.* **2006,** *97* (17), 170201.

(2) Pastewka, L.; Robbins, M. O. Contact between Rough Surfaces and a Criterion for Macroscopic Adhesion. *Proc. Natl. Acad. Sci.* **2014,** *111* (9), 3298-3303.

(3) Gigli, L.; Vanossi, A.; Tosatti, E. Modeling nanoribbon peeling. *Nanoscale* **2019,** *11* (37), 17396-17400.

(4) Gigli, L.; Kawai, S.; Guerra, R.; Manini, N.; Pawlak, R.; Feng, X.; Müllen, K.; Ruffieux, P.; Fasel, R.; Tosatti, E.; Meyer, E.; Vanossi, A. Detachment Dynamics of Graphene Nanoribbons on Gold. *ACS Nano* **2019,** *13* (1), 689-697.

(5) Ouyang, W.; Mandelli, D.; Urbakh, M.; Hod, O. Nanoserpents: Graphene Nanoribbon Motion on Two-Dimensional Hexagonal Materials. *Nano Lett.* **2018,** *18* (9), 6009-6016.

(6) Lin, K.; Zhao, Y.-P. Mechanical Peeling of Van der Waals Heterostructures: Theory and Simulations. *Extreme Mech. Lett.* **2019,** *30*, 100501.

(7) Leven, I.; Azuri, I.; Kronik, L.; Hod, O. Inter-Layer Potential for Hexagonal Boron Nitride. *J. Chem. Phys.* **2014,** *140* (10), 104106.

(8) Leven, I.; Maaravi, T.; Azuri, I.; Kronik, L.; Hod, O. Interlayer Potential for Graphene/h-BN Heterostructures. *J. Chem. Theory Comput.* **2016,** *12* (6), 2896-905.

(9) Maaravi, T.; Leven, I.; Azuri, I.; Kronik, L.; Hod, O. Interlayer Potential for Homogeneous Graphene and Hexagonal Boron Nitride Systems: Reparametrization for Many-Body Dispersion Effects. *J. Phys. Chem. C* **2017,** *121* (41), 22826-22835.

(10) Ouyang, W.; Azuri, I.; Mandelli, D.; Tkatchenko, A.; Kronik, L.; Urbakh, M.; Hod, O. Mechanical and Tribological Properties of Layered Materials under High Pressure: Assessing the Importance of Many-Body Dispersion Effects. *J. Chem. Theory Comput.* **2020,** *16* (1), 666-676.

(11) Steele, W. A. The Physical Interaction of Gases with Crystalline Solids: I. Gas-Solid Energies and Properties of Isolated Adsorbed Atoms. *Surf. Sci.* **1973,** *36* (1), 317-352.

(12) Griffith, A. A.; Taylor, G. I. VI. The Phenomena of Rupture and Flow in Solids. *Philosophical Transactions of the Royal Society of London. Series A, Containing Papers of a Mathematical or Physical Character* **1921,** *221* (582-593), 163-198.

(13) Bartlett, M. D.; Croll, A. B.; King, D. R.; Paret, B. M.; Irschick, D. J.; Crosby, A. J. Looking Beyond Fibrillar Features to Scale Gecko-Like Adhesion. *Adv. Mater.* **2012,** *24* (8), 1078-1083.
